# Beyond words: Parental perceptions on human papilloma virus vaccination recommendations and its impact on uptake

**DOI:** 10.1016/j.pmedr.2021.101596

**Published:** 2021-10-11

**Authors:** Teresa K.L. Boitano, Casey Daniel, Young-il Kim, J. Michael Straughn, Sylvia Peral, Isabel Scarinci

**Affiliations:** a Division of Gynecologic Oncology, Department of Obstetrics & Gynecology, O’Neal Comprehensive Cancer Center, University of Alabama at Birmingham, Birmingham, AL, USA; b Mitchell Cancer Institute, University of South Alabama, Mobile, AL, USA; c Division of Preventive Medicine, Department of Medicine, O’Neal Comprehensive Cancer Center, University of Alabama at Birmingham, AL, USA

**Keywords:** HPV, Vaccination, Provider recommendations, Cervical cancer prevention, Patient education, Vaccine uptake

## Abstract

The goal was to evaluate how provider recommendations regarding Human Papillomavirus (HPV) vaccination impact uptake in a rural setting. The study used a cross-sectional, population-based design with cluster sampling to survey parents/guardians of children ages 9 to 18 in 2019 in Alabama. In addition to demographics/knowledge questions, participants were asked about type of healthcare provider recommendation and impressions they had from this interaction. The primary outcome variable was the result of the healthcare provider recommendation with options including: child got vaccinated day of recommendation, HPV vaccination was scheduled, or HPV vaccination was not scheduled. Bivariate analysis and multinomial logistic regression were performed. Of the 358 respondents, approximately 40% indicated receiving a recommendation from a provider to vaccinate their child. Age of the parent, race/ethnicity, educational attainment, and health insurance coverage were similar across groups. Female parents were more likely to report receiving HPV vaccination recommendation than males (44.1% vs. 23.3%; p = 0.009). The type of provider recommendation was not significantly associated with HPV vaccination uptake. The impression from the recommendation of HPV vaccination being “important” was significantly associated with the child being vaccinated that day (OR = 7.31, 95% CI = 2.20–24.3) as well as scheduling HPV vaccination (OR = 3.17, 95% CI 1.01–9.92). Parents who got the impression that “there was no hurry” were less likely to vaccinate their child that day (OR = 0.23, 95% CI = 0.09–0.59). Provider recommedation is well-established as a significant predictor of HPV vaccination, these findings indicate that how the recommendation is perceived may play a more important role in HPV vaccination uptake.

## Highlights

• Provider recommendations significantly improve HPV vaccination rates.
• The emphasis a provider gives with a recommendation is important.
• Stressing the importance of same-day vaccination increases uptake.

## 1 Introduction

Human papillomavirus (HPV) remains the most common sexually transmitted infection in the United States (U.S.). Persistent HPV infection is responsible for approximately 90% of anal and cervical cancers, 70% of vaginal/vulvar cancers, 60% of penile cancers, and up to 70% of oropharyngeal cancers (Saraiya et al., 20152015, Timbang et al., 2019). In 2018, there were 45,000 new cases of HPV-associated cancers diagnosed in the U.S. (Division of Cancer Prevention and Control CfDCaP. How Many Cancers Are Linked with HPV Each Year, 2020).

The HPV vaccine protects against new HPV infection and consequently most HPV-associated cancers (Drolet et al., 2019, Lei et al., 2020). The Centers for Disease Control and Prevention (CDC) along with the Advisory Committee on Immunization Practices recommend that all adolescents get two doses of the vaccine between ages 11–12 years old, but vaccination can start as early as 9 years old with catch-up through 26 years old. When backed by shared-decision making, the vaccine has also recently been approved up to age 45 in individuals who were not previously adequately vaccinated (FDA approves expanded use of Gardasil 9 to include individuals 27 through 45 years old [press release], 2018). A recent study looking at over one million women in Sweden found that the quadrivalent HPV vaccine was associated with a “substantially reduced risk of invasive cervical cancer,” further confirming established evidence of the vaccine’s effectiveness (Lei et al., 2020). Despite this, the percentage of adolescents aged 13–17 with up-to-date (UTD) HPV vaccination in the U.S. is around 54% (Elam-Evans et al., 2020) which is far below the Healthy People 2020 goal of 80% of adolescents with an UTD HPV vaccine status (Services USDoHaH, 0000). UTD vaccination entails completing 2–3 shots in the vaccine series depending on age requirements. In Alabama, rates of UTD vaccination are even lower (around 47%) (Elam-Evans et al., 2020). This low level of coverage translates into a large missed opportunity of decreasing the risk of precancers, cervical cancer, oropharyngeal cancer, genital warts, and other anogenital cancers.

Research exploring reasons for low HPV vaccination rates have identified numerous facilitators and barriers. These include parent-specific and provider-specific barriers. Studies have demonstrated that parental barriers to vaccination include lack of education/knowledge, safety concerns, absence of provider recommendation, and concerns about promoting sexual behavior (Holman et al., 2014, Dilley et al., 2018). Provider-level barriers include lack of time to educate and discuss the vaccine, being uncomfortable discussing sex, knowledge gaps, vaccination access issues, and financial concerns (Holman et al., 2014, Dilley et al., 2018). Regarding facilitators, previous studies have consistently shown that a healthcare provider recommendation is the strongest predictor of HPV vaccination uptake (Gilkey et al., 2016, Hswen et al., 2017), even more so than other influencing variables such as access to care, race, belief system, or vaccination understanding (Kester et al., 2013, Lubker and Lynge, 2019). In rural settings, barriers to HPV vaccination include concern about vaccine safety and perceived need (Dilley et al., 2018, Cartmell et al., 2018). Regardless of rurality, parents have frequently expressed concern about HPV vaccination and its association with sex and a lack of knowledge about the vaccine (Suryadevara et al., 2021). Most studies to date have been performed in urban centers (Newman et al., 2018) but around 20% of the population in the U.S. is rural (Staff, 2017).

Although provider recommendation is the most significant predictor, previous work has determined that how the HPV vaccine recommendation is given is important and an influencing factor in itself. When providers give a high-quality recommendation, parents are more likely to vaccinate their child (Suryadevara et al., 2021, Ylitalo et al., 2013, Rahman et al., 2015). High-quality recommendations have been defined as having a strong HPV vaccine endorsement, a prevention message, and urgency in getting the vaccine. Recent data has shown some promise that when educated about the potential cancer prevention benefits of the vaccine, parents are more likely to vaccinate their child (Suryadevara et al., 2021). Despite this, up to 50% of parents reported no HPV vaccination recommendation and among those who did receive a recommendation, only about one-third were considered high-quality (Gilkey et al., 2016, Gilkey et al., 2018) suggesting that examination of how these recommendations are perceived by parents should be further examined. Therefore, the goal of this study was two-fold: (1) to examine the impact of healthcare providers’ recommendations on parents’ decision to vaccinate their children in a high-risk, rural region of the U.S.; and (2) to examine the impact of how parents received these recommendations (e.g. “it is important,“ “there was no hurry“) on HPV vaccination uptake/scheduling.

## 2 Materials and methods

This was a cross-sectional, population-based study using cluster sampling in which interviewers were deployed to obtain a representative sample from all nine census tracts in a rural county in Alabama (Escambia County). Parents/guardians of children ages 9–18 years old were interviewed between 9/2019 and 12/2019 and analyzed in 2020. Interviews were anonymous and participation was voluntary. If participants had more than one child in this age range, they were asked to think about the child with the closest birthday month when asked specific questions about HPV vaccination. There was no evaluation based on sex of parent in combination with the sex of the child. Inclusion criteria were being a resident of Escambia County and being the parent or guardian of a child between the ages of 9 and 18.

A 16-page questionnaire was developed based on questions from the Behavioral Risk Factor Surveillance System (BRFSS) ((CDC) CfDC, 2016) and other instruments. Questions were focused on healthcare status, access to care, reported HPV vaccination recommendation by health care providers and how these recommendations were perceived by parents (impression) along with other variables of interest. Specifically, individuals were surveyed regarding the recommendation style and impression of provider recommendation regarding HPV vaccination (Table 1). With regard to provider’s recommendations, parents/guardians were first asked: “Thinking about your (inserted age of the child) year-old child, has a doctor or healthcare professional ever advised you to get him/her vaccinated against HPV” (Victory et al., 2019)? Respondents were given the options of “yes”, “no”, or “I don’t know/I am not sure.” If they answered “yes,” they were then asked: “Thinking of your (inserted age of the child) year-old child, how did the doctor or healthcare professional recommend that he/she be vaccinated against HPV?” Response options were based on our previous qualitative/quantitative assessments across the state, as well as other studies (Dilley et al., 2018, Gilkey et al., 2016), and they were given the option to endorse more than one response (see Table 1 for response options). Parents/guardians were then asked: “Thinking of about your (inserted age of the child) year-old child, when the doctor or health professional recommended the HPV vaccine for him/her, what impression(s) did you get from them about the vaccine?” Response options included: “it was important”, “it was urgent”, “it was optional”, “it could wait/there was no hurry”, and “I do not know/I am not sure.” Like the previous question, respondents could choose more than one answer. Parents had to specify the outcome variable which included: child got vaccinated the day of the recommendation, HPV vaccination was scheduled, and HPV vaccination was not scheduled. Ten parents/guardians indicated that they did not know or were not sure in their responses, and they were excluded from the analyses. Demographic characteristics of the study participants were summarized by descriptive statistics. Bivariate associations of provider recommendations and impression from the recommendation with HPV vaccination uptake were measured by performing Chi-square test. Stepwise logistic regression models were evaluated to identify which provider recommendations or impression from the recommendation were associated with HPV vaccination uptake adjusted by age of the child. All statistical analyses were performed using SPSS v.25 and SAS v.9.4. This study underwent IRB approval at our institution. Funding was provided from both the O'Neal Comprehensive Cancer Center and the Mitchell Cancer Insitute. Final results were determined to be statistically significant when the accompanying statistical test yields a probability of 0.05 or less.

**Table 1:** Sample of Survey Questions.

Question:	Possible response(s):
Thinking about your (insert age) year-old child, has a doctor or health professional ever advised you to get him/her vaccinated against HPV?	- Yes - No - I don’t know/I am not sure - Refused
Thinking about your (insert age) year-old child, how did the doctor or health professional recommend that he/she be vaccinated against HPV? (Check all that apply)	- They expressed their personal belief in the importance of the HPV vaccine - They said that experts (like the American Academy of Pediatrics) agree that getting the HPV vaccine is important for all children - They said that there have been less cases of HPV-related diseases since the HPV vaccine was introduced - They said that the HPV vaccine is very safe - They said that they have/would give their own children the HPV vaccine - They simply stated that your child should get the HPV vaccine - They said that the HPV vaccine prevents a number of HPV-related cancers - They said that the HPV vaccine is very effective - They asked if you had any questions or concerns about the HPV vaccine - Other (specify): - I don’t know/I am not sure - Refused
Thinking about your (insert age) year-old child, how much did you trust his/her provider’s recommendation?	- Completely trusted their recommendation - Somewhat trusted their recommendation - Did not trust their recommendation at all - I don’t know/I am not sure - Refused
Thinking about your (insert age) year-old child, when the doctor or health professional recommended the HPV vaccine for him/her, what impression(s) did you get from them about the vaccine? (Check all that apply)	- It was important - It was urgent - It was optional - It could wait/there was no hurry - I don’t know/I am not sure - Refused
Thinking about your (insert age) year-old child, what was the immediate outcome of this recommendation?	- Child got HPV vaccine that day - Child did not get HPV vaccine that day, but scheduled it - Child did not get HPV vaccine and did not schedule one - I don’t know/I am not sure - Refused

## 3 Results

A total of 368 parents/guardians who had at least one child between the age of 9 and 18 were included in the final sample, for a response rate of 83.4% (number of surveys completed/ number of eligible parents approached). Of these, 149 (40.5%) indicated that a doctor or healthcare provider advised them to vaccinate their child against HPV, 209 (56.8%) indicated that they had not received such recommendation, and 10 (2.7%) reported that they did not know or they were unsure. There were no significant differences between parents/guardians who indicated receiving such recommendation compared with those who did not with regard to parent/guardian’s age, race/ethnicity, educational attainment, the child having a regular source of care, and having health insurance coverage (Table 2). However, female parents were more likely to report receiving HPV vaccination recommendation than male parents (44.1% vs. 23.3%; p = 0.009), as were parents who had older versus younger children (14.2 vs. 12.1 years old; p = 0.0001).

**Table 2:** Demographic Profile of the Sample by Provider Recommendation of HPV Vaccination*

	Yes (N = 149)	No (N = 209)	p-value
Age of parent/guardian (years)	40.5 ± 7.7 **	38.8 ± 9.8**	0.08
Age of the child (years)	14.2 ± 2.7 **	12.1 ± 2.9**	**<0.0001**
Race/Ethnicity White African American American Indian Mixed race Hispanic	60 (41.7%) 73 (41.5%) 10 (47.6%) 2 (33.3%) 4 (36.4%)	84 (58.3%) 103 (58.5%) 11 (52.4%) 4 (66.7%) 7 (63.6%)	0.96
Sex Male Female	10 (23.3%) 139 (44.1%)	33 (76.7%) 176 (55.9%	**0.009**
Educational Attainment Less than high school High school or GED Some college College	13 (33.3%) 57 (39.6%) 53 (43.8%) 26 (48.1%)	26 (66.7%) 87 (60.4%) 68 (56.2%) 28 (51.9%)	0.5
Child having a regular source of healthcare	147 (41.8%)	205 (58.2%)	0.7
Child having health insurance coverage	146 (41.6%	205 (58.4%)	0.9

Bolded text in the p-value section implies significance.

*10 participants responded “don’t know/not sure” and were excluded from the analysis

** Mean ± Standard Deviation

There was no significant association between type of provider recommendation (i.e. “provider said that the HPV vaccine is very safe” or “provider stated he/she would vaccinate his/her own children”) and HPV vaccination uptake or scheduling in the bivariate analysis (Table 3). However, the impression from the recommendation of HPV vaccination being “important” was significantly associated with the child getting vaccinated that day (OR = 7.31; 95% CI 2.20–24.3) as well as scheduling HPV vaccination as compared with not vaccinating/scheduling in the multinomial logistic regression (OR = 3.17; 95% CI 1.01–9.92) (Table 4). Parents who got the impression from their provider that “there was no hurry” were less likely to vaccinate their child compared to those who vaccinated the child the day of the recommendation (OR = 0.23; 95% CI 0.09–59). Age of the child had no significant association as to whether the parents got their child vaccinated or not (OR = 1.03; 95% CI 0.87–1.21).

**Table 3:** Bivariate Analysis of Provider Recommendations and Parent/Guardian Impression from the Recommendation by HPV vaccination uptake.

	Child got HPV vaccine that day	Child did not get HPV vaccine, but scheduled it	Child did not get HPV vaccine, but did not schedule it	p-value
**Provider recommendation:** - Provider expressed personal belief on importance of the vaccine - Experts agree on importance of vaccine - Less cases of HPV since vaccine - HPV vaccine safe - Provider would give own child HPV vaccine - Your child should get vaccinated - HPV vaccine prevents HPV- related cancers - HPV vaccine is very effective - Provider asked if parent had questions	n = 147			
	47 (61.8%) 65 (85.5%) 35 (46.1%) 60 (79.0%) 47 (61.8%) 51 (67.1%) 59 (77.6%) 59 (77.6%) 64 (84.2%)	23 (59.0%) 33 (84.6%) 15 (38.5%) 25 (64.1%) 24 (61.5%) 25 (64.1%) 27 (69.2%) 30 (76.9%) 30 (76.9%)	19 (59.4%) 22 (68.8%) 13 (40.6%) 21 (65.3%) 14 (43.8%) 19 (59.4%) 23 (71.9%) 21 (65.6%) 25 (78.1%)	0.9 0.1 0.7 0.2 0.2 0.7 0.6 0.4 0.6
**Impression from the provider about the vaccine:** It was important It was urgent It was optional There was no hurry	71 (93.4%) 30 (30.5%) 48 (63.2%) 14 (18.4%)	33 (84.6%) 12 (30.8%) 29 (74.4%) 14 (35.9%)	20 (62.5%) 5 (15.6%) 27 (84.4%) 17 (53.1%)	**0.0003** **0.05** 0.07 **0.001**

*Percentages are based on yes versus no answer in each group. Bolded text in the p-value section implies significance.

**Table 4:** Multinomial Logistic Regression of Provider Recommendations and Parent/Guardian Impression from the Recommendation by HPV vaccination uptake.

	Child got HPV vaccine that day	Child did not get HPV vaccine, but scheduled it
**Impression from the provider about the vaccine:** It was important There was no hurry	n = 147	
	**7.31 (2.20**–**24.30)** **0.23 (0.09**–**0.59)**	**3.2 (1.0**–**9.9)** 0.5 (0.2–1.4)
**Age of the child**	1.03 (0.87–1.21)	0.9 (0.8–1.1)

*Reference category: Child did not get HPV vaccine that day, and did not schedule one

Values are odds ratios (95% Wald CI).

**Stepwise selection was applied with p-value < 0.1 to entry and p < 0.05 to stay

## 4 Discussion

The findings of this study emphasize the importance of how HPV vaccination recommendation is received by parents. In this sample of rural residents, recommendation style was not associated with the HPV vaccination uptake but how parents perceived these recommendations was. The average uptake for most routine vaccines in adolescents is 88–95% compared to the HPV vaccine at 51% (Walker et al., 2018) with drastic variation of uptake across the states. For instance, Rhode Island has a 78% HPV vaccination rate and ranks #1 in the United States, whereas, many of the southern, rural states (South Carolina, Mississippi, and Alabama) have vaccination rates around 50% placing them in the last quartile (Waldrop et al., 2017). Compared to urban U.S. populations, rural populations have a lower rate of HPV vaccianation as noted above and a higher incidence of HPV-related cancer (Peterson et al., 2020). Provider recommendation has been shown to be the most important variable associated with HPV vaccination uptake, and a number of efforts have been implemented to booster providers’ recommendation (Gilkey et al., 2016, Suryadevara et al., 2021, Newcomer et al., 2020). For instance, announcements, or brief statements assuming parents are ready to vaccinate, have been used for most early childhood vaccines with success (Brewer et al., 2017). A recently published study showed that utilizing specific communication techniques (i.e. announcementns) allow for more efficient, effective, and shorter discussions (Fenton et al., 2021) but ambiguous language is associated with lower vaccination rates (Fenton et al., 2018).

This study further demonstrated that what providers say may be less important than how they actually say it. When the parent/guardian got the impression that the vaccine was important, the child was more likely to get vaccinated the day of the visit. Furthermore, when parents got the impression that “there was no hurry” to vaccinate their child, they were less likely vaccinate their child at all. This proposes the concept that if parents are presented with the HPV vaccine as a necessary requirement this will normalize it to be considered as part of routine care and vaccination rates should increase. This further endorses previous recommendations from the CDC to encourage parents that the HPV vaccine is important, emphasize the cancer prevention component, and recommending same-day vaccination (Gilkey et al., 2016).

The topic of how a provider should approach HPV vaccination counseling is still widely discussed. We attempted to evaluate if the recommendation style, i.e. “the vaccine is safe” or “the provider would get their own child vaccinated,” made a significant impact on parents’ uptake of the vaccine. Research has demonstrated that placing an emphasis on the preventive measures of the vaccine, i.e. “decreasing the risk of cervical cancer,” has shown to motivate parents (Gilkey et al., 2016, Gilkey et al., 2018). Other methods such a personal endorsement, noting the importance of a timely vaccine, and comparing it to other routine vaccines have all been employed to integrate the vaccine into routine healthcare. Interestingly, this study did not show that any one particular recommendation was more likely to influence parental decision-making. Instead, it was found that it was more significant that the provider stress the importance of getting the vaccine without delay.

Given the racial/ethnic disparities in cervical cancer incidence and mortality (Yoo et al., 2017), there have been concerns in the past that that non-Hispanic blacks were less likely to get the recommendation to vaccinate their child than non-Hispanic whites but trends have shown this gap to be narrowing (Ylitalo et al., 2013, Burdette et al., 2017). In some regions of the United States, adolescent minorities have higher HPV vaccination initiation rates than their non-Hispanic white counterparts, but then have a lower completion rate with the full vaccine series (Spencer et al., 2019). Recommendation rates were similar regardless of race/ethnicity, age, or insurance coverage in this study. Female parents were more likely to report receiving the recommendation compared with male parents. Ten percent of parents involved in the study were male, meaning this is an important demographic for providers to be aware of in order to not avoid this conversation. Furthermore, parents with older children were more likely to receive the recommendation. Given the CDC recommends HPV vaccination starting as early as 9 years-old, and that if a child is vaccinated < 15 years-old they only need two instead of three doses, providers should emphasize vaccination at the youngest-recommended age possible.

Limitations of this study included it being performed in a single state and rural county which means findings may not be as generalizable to all of the United States. Given it is a self-reported survey, it also may include some recall bias and unintentional misrepresentation of vaccination status. Strengths of this study include a questionnaire based on previously proven surveys, a good response rate, and being a population-based survey. Most HPV vaccination data at the population level in the United States rely on telephone-based surveys (e.g., Behavioral Risk Surveillance System, National Health Interview Survey) and limited representation of rural residents. Further, when rural residents are included most studies limit to the residents in the towns and are not inclusive of the entire county. This study included a balanced representative sample from all nine census tracts in a rural county.

## 5 Conclusion

The amount of strong evidence demonstrating that provider recommendations significantly improve HPV vaccination rates has created optimism and increased efforts in this area. The findings of this study emphasize the importance of the impression that the provider gives. Providers stressing the importance of the HPV vaccine increased same-day vaccination uptake while a “lack of hurry” impression led to decreased same-day vaccination. There was no indication that specific phrases altered vaccination rates. Instead, the sole difference was made with provider rhetoric emphasizing importance. This potentially allows providers to spend less time over-explaining the HPV vaccination and instead provide the parents with reassurance that it is an important part of routine healthcare. Ultimately, the goal is to continue to advance communication practices with parents, guardians, and patients in order to improve cancer prevention through the simple, yet effective, mechanism of vaccination.

### CRediT authorship contribution statement

**Teresa K.L. Boitano:** Data curation, Writing – original draft, Writing – review & editing. **Casey Daniel:** Conceptualization, Methodology, Resources. **Young-il Kim:** . **J. Michael Straughn:** Writing – review & editing. **Sylvia Peral:** Data curation, Methodology, Writing – review & editing. **Isabel Scarinci:** Supervision, Conceptualization, Methodology, Resources, Writing – review & editing.

## Declaration of Competing Interest

The authors declare that they have no known competing financial interests or personal relationships that could have appeared to influence the work reported in this paper.
